# Exploiting the power of stepwise intraoperative irrigant activation to maximize oval canal disinfection: an ex-vivo investigation

**DOI:** 10.1186/s12903-025-06493-2

**Published:** 2025-07-05

**Authors:** Mohammed Turky, Shaimaa Hamdy, Soha Elhady

**Affiliations:** 1 Department of Endodontics, Faculty of Dentistry, Minia University, Agricultural Misr-Aswan Road, Minia, Egypt; 2 Department of Endodontics, Faculty of Dentistry, Sphinx University, Assiut, Egypt; 3 Department of Oral Diagnosis, Oral Medicine and Periodontology, Faculty of Dentistry, Nahda University, Beni Swef, Egypt; 4 Department of Medical Microbiology & Immunology, Faculty of Medicine, Ain Shams University, Cairo, Egypt

**Keywords:** Endodontics, *Enterococcus faecalis*, Irrigation, Root canal treatment, Stepwise intraoperative activation

## Abstract

**Objectives:**

To evaluate the antibacterial effectiveness of stepwise intraoperative activation of the root canal irrigant, both alone and when combined with conventional ultrasonic activation, compared to the traditional syringe irrigation method in oval root canals.

**Methods:**

Sixty single-rooted maxillary second premolars were selected according to specific criteria. After creating an access opening and root canal patency in all samples, a cycle of autoclave sterilization was conducted. *Enterococcus faecalis* suspension was inoculated into the root canals and incubated at 37 °C for 21 days. After bacterial contamination, the specimens were assigned to four experimental groups (*n* = 10 per group): conventional syringe irrigation (CSI); stepwise intraoperative activation (SIA), consisting of the ultrasonic activation of 2 mL of 5.25% NaOCl solution for 20 s following irrigation between instrumentation files; conventional ultrasonic irrigation (CUI), consisting of the passive intermittent ultrasonic activation of 2 mL of 5.25% NaOCl in three successive cycles of 20 s each at the end of the mechanical instrumentation; and stepwise intraoperative activation plus conventional ultrasonic irrigation (SIA + CUI), consisting of a combination of SIA and CUI; in addition to positive control; and negative control groups (*n* = 10). At the end of the chemo-mechanical preparation, a bacterial sampling was conducted to determine the number of colony-forming units per mL (CFU//mL), and the outcomes were compared with one-way ANOVA and Games-Howell post hoc test with the significance level set at 5%.

**Results:**

None of the tested irrigation protocols were able to fully eliminate the intracanal bacterial infection. However, all protocols succeeded in reducing the bacterial burden within the oval root canals considerably (*p* <.05). The combination of SIA and CUI achieved the greatest bacterial reduction (1.85 ± 0.99), followed by the CUI (3.00 ± 0.13), SIA (3.54 ± 0.26), and CSI (4.29 ± 0.16) groups, respectively, with significant differences between them (*p* <.05).

**Conclusions:**

Stepwise intraoperative irrigant activation was able to reduce the bacterial load in the oval root canals significantly compared to the basic chemo-mechanical preparation, with maximal disinfection achieved when combined with conventional ultrasonic irrigation.

**Clinical relevance:**

The complex morphology of oval root canals presents significant challenges for effective disinfection during root canal treatment. To optimize the disinfection process, it would be essential to implement supplementary techniques in conjunction with standard root canal preparation. Employing a stepwise intraoperative irrigation activation method can enhance disinfection efficacy in these convoluted anatomies, particularly when integrated with traditional ultrasonic irrigation, potentially leading to improved treatment outcomes.

**Clinical trial number:**

Non-applicable. Conducting the current experiment was limited to the approval of the local Research Ethics Committee at the Faculty of Dentistry, Minia University, Egypt (Committee No. 105, registration No. 903).

**Supplementary Information:**

The online version contains supplementary material available at 10.1186/s12903-025-06493-2.

## Introduction

Bacterial infection plays an indispensable role in the pathogenesis of pulp and periapical diseases and is considered one of the major reasons for endodontic failures [1]. Consequently, root canal treatment aims to eliminate infection and prevent the re-entry of microorganisms into the root canal system [2]. In an effort to implement clinically effective treatments and preventive strategies, the pathobiology of root canal infections has received a lot of attention throughout the last few decades [2]. Despite the rapid progress in modern endodontics regarding mechanical instruments and disinfection protocols, total eradication of root canal infection is still impossible [3]. The reasons behind this fact lie in the presence of bacteria in complex microbial communities known as biofilm structures, which are made up of a wide range of bacteria with varying ecological requirements and potential for pathogenicity [4]. Apart from providing bacteria with a strong defense against the host’s immune system, the biofilm community increases the bacteria’s resistance to a range of disinfectants [4]. Moreover, the intricate root canal morphologies, including fins, ramifications, isthmus, and irregular cross-sections, add significant complexity to root canal disinfection [5–7].

Based on their cross-sections, root canals can be categorized as round, oval, long oval, flattened, or irregular. Oval root canals have a buccolingual dimension twice the mesiodistal dimension, whereas long oval canals have a buccolingual dimension two to four times the mesiodistal dimension [8, 9]. Disinfection of such configurations poses a challenge due to the hard-to-reach areas that increase the likelihood of forming bacterial aggregations able to cause treatment failure [10–12].

Although root canal instrumentation is crucial for reducing intraradicular bacteria [13, 14], mechanical instrumentation of oval root canals using either manual or contemporary automated nickel-titanium (NiTi) instruments often leaves unprepared buccal and lingual extensions or recesses, which may harbor debris, necrotic pulp remnants, and bacterial biofilms, resulting in inadequate cleaning and disinfection of the root canals [15, 16]. Therefore, hybrid instrumentation comprising both engine-driven instruments and manual instrumentation, such as using Hedstrom files, has been proposed to improve the preparation of oval root canals [17, 18]. Additionally, irrigation is a fundamental adjunct to mechanical instrumentation for better debridement and disinfection of the root canals, particularly for the areas beyond the reach of the mechanical instruments [19–21]. However, it is well-known that conventional syringe irrigation often leaves certain areas of the root canal system covered with smear layers, debris, and residual bacteria [22]. To address this issue, several additional techniques have been developed. One of these techniques is passive ultrasonic irrigation, which has recently revived the use of ultrasonics in endodontics [23]. Passive ultrasonic irrigation is typically utilized to activate the irrigant at the end of chemo-mechanical preparation, facilitating the debridement and disinfection of difficult-to-reach locations and complex anatomies [5, 24, 25]. A recent systematic review of randomized clinical trials and meta-analysis revealed that the antimicrobial effectiveness of ultrasonic irrigation is superior to that of the conventional syringe irrigation method [26]. Nevertheless, it is unable to render root canals completely free of bacteria, potentially compromising treatment outcomes [27]. Therefore, further disinfection protocols should be implemented to ensure maximum disinfection of the root canals.

Stepwise intraoperative activation involves the activation of the irrigant (sodium hypochlorite solution [NaOCl]) between mechanical files to enhance the effectiveness of the irrigation protocol [28]. Although the predictability of such a technique in smear layer and debris removal has been reported [28], no information is currently available regarding its antimicrobial efficacy either alone or in conjunction with conventional ultrasonic irrigation. Therefore, the present study aimed to compare the antibacterial effectiveness of stepwise intraoperative activation of the irrigant alone and in combination with conventional ultrasonic activation to the conventional syringe irrigation method in oval root canals. The hypothesis tested would be that there is no significant difference in the antibacterial effectiveness among the various irrigation protocols in oval canals.

## Materials and methods

### Ethical considerations

The present study was conducted in strict alignment with the ethical principles established by the Declaration of Helsinki, which emphasizes the importance of safeguarding the rights and welfare of research participants. Prior to the commencement of the study, formal approval was obtained from the Research Ethics Committee at the Faculty of Dentistry, Minia University, Egypt, under registration no. 903. This approval process ensured that our research design and methodologies complied with the relevant ethical guidelines and regulations governing human subject research.

To further ensure ethical compliance and participant safety, written informed consent was meticulously gathered from all individuals participating in the study. This consent process involved providing detailed information about the nature of the research, the procedures involved, potential risks, and the benefits of participation, allowing participants to make an informed decision regarding their involvement.

### Sample size calculation

To determine the appropriate sample size, a pilot study was conducted, which included 5 teeth for each group. To ensure the study’s statistical validity, a power analysis was performed using G*Power software 3.1.9.7 (Heinrich Heine University in Düsseldorf, Germany) to calculate the optimal sample size needed to effectively test the null hypothesis. An alpha (α) level of 0.05, which reflects a 5% threshold for statistical significance, was selected, and a beta (β) level of 0.05, resulting in a desired power of 95%. For the One-Way ANOVA test applied in this study, the effect size (f) was calculated to be 0.99, signifying a large effect. Based on these parameters, the minimal sample size required was determined to be 30 teeth, with each group comprising 5 teeth.

This in vitro study aimed to evaluate the efficacy of various disinfection protocols in achieving bacterial reduction within oval root canals, a common concern in endodontics. The calculated effect size of 0.99 signifies a robust and clinically meaningful difference between the groups or interventions assessed. Such a large effect size suggests that the disinfection protocols employed had a profound and impactful influence on minimizing the bacterial load present in the root canals, reinforcing its potential application in clinical endodontic practice. Moreover, the study included a detailed analysis of the 95% confidence interval (CI) associated with the effect size, which offers important insights into the statistical precision and reliability of the observed results. A narrow CI would indicate a high level of certainty regarding the effectiveness of the disinfection methods, implying that the results are consistent and reproducible. In contrast, a wider CI could denote variability within the data that may necessitate further exploration to understand the underlying factors contributing to the differences observed. The combination of a substantial effect size and robust confidence intervals highlights not only the strength of the evidence presented but also the potential clinical relevance of these findings in the field. This suggests that implementing the evaluated disinfection protocols could significantly enhance treatment outcomes for patients undergoing endodontic procedures, potentially leading to improved oral health and reduced incidence of endodontic failures.

### Sample selection

A total of 60 freshly extracted human intact single-rooted maxillary second premolars with fully formed apices, extracted with minimal trauma for either orthodontic or periodontal reasons, were collected from healthy patients aged 20–40 years old at the outpatient clinic, Faculty of Dentistry, Minia University, Egypt. Pair matching for tooth morphology, pulp space anatomy, and volume was achieved using 3D imaging (Papaya 3D plus, Genoray, Gyeonggi-do, Korea). All selected teeth had single root canals classified as Vertucci’s type I [29] with an oval cross-section, where the buccolingual dimension was twice the mesiodistal dimension, an equivalent root canal curvature angle of less than 10° as determined by Schneider’s method [30], and a radius of less than 5 mm. To ensure comparable working lengths, teeth were selected where the canal terminus aligned with the root apex. Teeth with caries, pre-existing restorations, previous endodontic treatment, calcifications, internal or external root resorption, or evidence of cracks or fractures were excluded and replaced with ones that met the inclusion criteria for the present study. The selected samples underwent ultrasonic scaling to remove any attached hard or soft deposits, followed by disinfection in a 5.25% NaOCl solution (Omez, Phar Omez, Pharaonic Pharmaceuticals, Egypt) for 30 min. Subsequently, the samples were stored in a 0.1% thymol suspension (Formula e Acao, São Paulo, SP, Brazil) at 4^o^C for a maximum of one month until use.

### Sample preparation

Standardized traditional access cavities were established using a sterile round diamond bur (# 801 − 014; Komet Dental, Braseler GmbH & Co. KG, Lemgo, Germany) and refined using a sterile safe-ended tapered carbide fissure bur (Komet H33L; Komet Dental, Braseler GmbH & Co. KG, Lemgo, Germany). To ensure sterile procedures, all burs used for the access cavity preparations were autoclaved at 121º C for 15 min and mounted on a sterile high-speed handpiece with water spray. Subsequently, the pulp chamber was irrigated and soaked in 5.25% NaOCl for 5 min. Root canal patency was checked using a sterile stainless-steel K-file ISO size 10 (Dentsply, Maillefer, Ballaigues, Switzerland). Teeth with non-negotiable root canals or those with initial apical diameters larger than size 20 based on the first apical binding file were discarded and replaced with others that fulfilled the selection criteria of the current research.

The occlusal reduction was performed to standardize all the teeth’s lengths at an average of 19 mm. The working length was determined visually by inserting a sterile stainless-steel K-file ISO size 10 until it was observed through the apical foramen. Then, 1 mm was subtracted from this measurement. To facilitate efficient bacterial inoculation throughout the entire canal length and ensure consistent bacterial distribution across the study samples, a manual glide path was achieved up to a sterile K-file ISO size 20 (Dentsply, Maillefer, Ballaigues, Switzerland) under copious irrigation with 5 mL of 5.25% NaOCl for 1 min, which was then inactivated with 10% sodium thiosulfate. Finally, the root canals received 5 mL of saline irrigation for 1 min, followed by 5 mL of ethylenediaminetetraacetic acid (EDTA) (Prevest DenPro Limited, Jammu & Kashmir, India) for 1 min in an attempt to effectively eliminate the smear layer generated during initial root canal enlargement that can adversely influence biofilm attachment to canal walls. A final rinse of saline solution with the same volume and for the same duration was performed to address the residual effect of the EDTA solution.

To prevent bacterial ingress, the root surface was coated with nail varnish, leaving the root apex exposed while maintaining canal patency. This setup allows for the closed systems that create the vapor-lock phenomenon, which may affect the disinfection in the apical area. In addition, the access cavity was sealed with a temporary restoration (Cavit G, 3 M ESPE, Neuss, Germany).

To ensure the absence of any residual infection, all samples were placed inside 20 mL Falcon tubes filled with distilled water and autoclaved at 121 °C for 15 min. For adequate sterilization, each sample was irrigated with 1 mL of sterile saline solution. Then, a small sterile stainless-steel H-file (ISO size 20) was used in a pumping motion along the canal walls, and a culture was taken using three sterile paper points size 20 (Dentsply, Maillefer, Ballaigues, Switzerland), which were inserted into the root canals sequentially for 1 min each. The samples were then plated onto brain-heart infusion agar plates and incubated for 21 days at 37 °C. Finally, the teeth were mounted vertically in acrylic resin and placed in Eppendorf tubes to facilitate handling and identification.

### Bacterial inoculation

The *Enterococcus faecalis* (*E. faecalis*) clinical isolate, identified as American-type Culture Collection [ATCC] 29212, was grown in brain-heart infusion (BHI) broth (Oxoid CM225) for 24 h at 37 °C. The bacterial concentration was adjusted to more than 10^8^ colony-forming units (CFU)/mL, and 1 mL of this broth was added to fresh BHI to create a suspension. Using sterile micropipettes, 10 µL of the *E. faecalis* suspension was added to each canal, and then the canal orifice was covered with sterilized cotton. A sterile K-file ISO size 20 (Dentsply, Maillefer, Ballaigues, Switzerland) was used to distribute the bacterial suspension uniformly up to the predetermined working length. Subsequently, the specimens were incubated in *E. faecalis* suspension (> 10^8^ CFU/mL) at 37 °C for 21 days, with the suspension being refreshed every 72 h. For each specimen, two samplings were taken: one prior to preparation and another after preparation [31].

### Bacterial sampling before chemo-mechanical preparation

This procedure aimed to ensure a consistent and homogeneous bacterial distribution across the different study samples, thereby potentiating the reliability of the findings. At the outset, a sterile saline solution was injected into the root canals and evenly distributed throughout the entire length of the root canal using a sterile K-file ISO size 20. Afterward, the root canal walls were scraped circumferentially using a sterile stainless-steel H-file ISO size 20 up to the working length, followed by the insertion of three successive sterile paper points size 20 into the root canals for one min each. The paper points were then removed using sterile tweezers and placed in sterile plastic tubes containing 1 mL of physiological saline solution. The tubes were vortexed for 30 s, and 20 µL aliquots were plated onto BHI agar plates after being serially diluted 10-fold in sterile saline solution. Finally, the plates were incubated for 48 h at 37 °C [13].

### Sample classification

After root canal contamination with *E. faecalis*, the teeth were allocated utilizing the stratification process. Teeth exhibiting similar anatomical features, dimensions, and age were assigned to the various study groups. including four experimental groups (*n* = 10) based on the irrigation protocol, along with one positive control group and one negative control group (*n* = 10), as follows:

- *Experimental group 1- Conventional syringe irrigation (CSI) group*: In this group (*n* = 10), root canals were instrumented using Hyflex CM rotary file systems (Coltene Whaledent, Cuyahoga Falls, OH, USA), a multi-file rotary system employed in a continuous rotation motion, mounted on a controlled torque electrical endodontic motor (TriAuto mini; J. Morita MFG, CORP, Japan). Only one file set was utilized for each canal, and all instruments were autoclaved before instrumentation at 121 °C for 20 min. After adjusting the speed and torque as per the manufacturer’s instructions, coronal flaring was achieved using a file size of 25/0.08, followed by the use of 20/0.04 and 25/0.04 files up to the predetermined working length. Subsequently, file size 20/0.06 was employed to enlarge the middle third of the root canal up to the working length, and finally, 30/0.04 and 40/0.04 instruments were utilized to the full working length. It is important to emphasize that this instrumentation sequence (25/0.08, 20/0.04, 25/0.04, 20/0.06, 30/0.04, 40/0.04) complied with the standard manufacturer-recommended sequence for HyFlex CM files, employing variable tapers to adapt to the root canal morphology, which was implemented in a previous study [32]. This approach not only aligns with the manufacturer’s guidance but also encourages clinicians to adjust protocols based on the specific morphology of each root canal.
  This particular sequence was intentionally selected to effectively navigate the anatomical complexities presented by oval root canals, especially within the challenging coronal and middle thirds of these canals. The process began with initial flaring using the 25/0.08 orifice opener to create an adequately shaped entrance, followed by preliminary shaping with 0.04 taper instruments. The introduction of the 20/0.06 file was a strategic choice aimed at enhancing circumferential dentin contact in the most oval segments of the canal. This selective use was designed to improve irrigation dynamics and disinfection efficacy while avoiding unnecessary enlargement in the apical third. Following this, a return to 0.04 taper instruments for apical finishing, culminating in up to a 40/0.04 file, was employed to maintain apical centering. This step is crucial as it limits canal transportation— a common complication that can arise during root canal procedures— and helps preserve the structural integrity of the dentin in the apical third. In addition to their featured cyclic fatigue resistance, which was precisely reported in well-standardized static and dynamic cyclic fatigue models under controlled bending stresses [33, 34], the exceptional flexibility and controlled memory characteristics of the HyFlex CM files were key factors that facilitated the safe application of this non-linear sequencing, especially in the context of intricate canal anatomies. In summary, this sequence effectively embodies an anatomy-driven shaping strategy and not only adheres to clinical objectives but also takes full advantage of the versatile application possibilities offered by the HyFlex CM system, ultimately leading to improved outcomes in challenging endodontic cases.
  Circumferential filing of the buccal and lingual extensions of the root canals, with three strokes for each surface, was performed using a stainless-steel H-file ISO size 40. Between mechanical instruments, root canals were irrigated with 2 mL of 5.25% NaOCl for 20 s, totaling 15 mL over 150 s throughout the instrumentation course, delivered through a 30-gauge side-vented closed-end irrigating needle (Fanta Dental Materials Co. Ltd., Shanghai, China) mounted on a plastic syringe and inserted 1 mm short of the working length using a back-and-forth movement while the irrigant was delivered. The final rinse was conducted as follows: 10 mL of 5.25% NaOCl for 2 min followed by 10 mL of 17% ethylenediaminetetraacetic acid (EDTA) for 2 min (Prevest DenPro Limited, Jammu & Kashmir, India), with intermediate rinses of the same volumes of saline solution for 2 min each. A final flush using saline solution was also performed to remove any residues of the employed irrigants.

- *Experimental group 2 - Stepwise intraoperative* activation *(SIA) group*: In this group (*n* = 10), the chemo-mechanical procedures mentioned earlier in the CSI group were repeated, with the addition of ultrasonic activation of 2 mL of 5.25% NaOCl solution for 20 s following irrigation between files, resulting in a cumulative volume of 30 mL for 5 min during mechanical instrumentation. This activation was done using an E1 Irrisonic tip (Helse Dental Technology, São Paulo, SP, Brazil) corresponding to size 20/0.01. The tip was sterilized in an autoclave prior to use at 121 °C for 20 min, then mounted on an ultrasonic unit with a power setting of 2 (20%), and passively inserted up to the level reached by the preceding mechanical file and the irrigating needle.
- *Experimental group 3 - Conventional ultrasonic irrigation (CUI) group*: In this group (*n* = 10), all the previously mentioned chemo-mechanical procedures in the CSI group were also followed in the CUI group. Furthermore, at the end of the chemo-mechanical preparation, passive intermittent ultrasonic activation of an additional fresh 2 mL of 5.25% NaOCl in three successive cycles of 20 s each was carried out using the same ultrasonic tip with the same power setting used in the SIA group. The tip was passively inserted up to 3 mm short of the working length in root canals flooded with NaOCl [35, 36]. This activation process was included as a part of the final irrigation protocol, following the delivery of 10 mL of 5.25% of NaOCl for 2 min, before proceeding with EDTA irrigation [27, 37, 38]. Compared to the standard CSI group, this group involved an extra 6 mL of 5.25% NaOCl, which was activated intermittently over a total time of 1 min.
- *Experimental group 4 - SIA + CUI group*: In this group (*n* = 10), root canal procedures as described in the CSI group were performed but enhanced with a combination of SIA and CUI, following the same methods mentioned in the SIA and CUI groups, respectively. In other words, during the root canal procedure, identical instrumentation and irrigation protocols were followed. This included a total of 15 mL of 5.25% NaOCl administered over 150 s during root canal instrumentation. The final rinse involved 10 mL of 5.25% NaOCl followed by 10 mL of 17% EDTA, each for 2 min, with both intermediate and final flushes of saline solution using the same volumes and duration. Moreover, stepwise intraoperative ultrasonic activation was applied, using 2 mL of 5.25% NaOCl for 20 s between mechanical files. This process resulted in an extra 15 mL of 5.25% NaOCl being delivered over 150 s. Conventional passive ultrasonic irrigation was also utilized, which included agitating an additional 6 mL of 5.25% NaOCl for 60 s.
- *Positive control (PC) group*: In this group (*n* = 10), the root canals were contaminated but not instrumented in order to assess bacterial viability throughout the experiment.
- *Negative control (NC) group*: In this group (*n* = 10), the root canals were instrumented but not contaminated to verify the sterility of the procedures.

Prior to bacterial sampling following root canal preparation, 10% sodium thiosulfate was used to inactivate NaOCl [39]. Owing to their impact on disinfection, several critical parameters were meticulously standardized. These parameters included the total exposure time to the disinfecting agents and the precise volumes of irrigation solutions employed that were carefully calibrated according to their specific group, while the number of in-and-out motions of the mechanical instruments, instrumentation time, and preparation size and taper were equivalent for all groups.

Furthermore, the concentrations of the diverse chemical agents employed in the disinfection process were adjusted with precision using iodometric titration. To maintain consistency and reliability across all groups, it was crucial to clarify that all root canal procedures were conducted by a single experienced endodontist (M.T.) who brings two decades of specialized expertise in the field of endodontics.

### Bacterial sampling after chemo-mechanical preparation

Following the completion of root canal preparation, the root canal walls were scraped circumferentially using a sterile stainless-steel H-file ISO size 20 up to the full working length. Subsequently, three successive sterile paper points size 40 (Dentsply, Maillefer, Ballaigues, Switzerland) were employed for bacterial culture, each for 1 min, using the same method as before the chemo-mechanical preparation. The number of colony-forming units per 1 mL was computed by counting the visible colonies on every plate and expressed as CFU/mL [13].

The evaluation process was conducted by an experienced examiner (S.E.) who was deliberately blinded to the specific study groups involved. This intentional blinding was crucial in minimizing biases, ensuring that the analysis of each sample was performed fairly and impartially, devoid of preconceived notions. By upholding this rigorous standard of objectivity, the integrity of the results was not only preserved but also strengthened. The commitment to an unbiased evaluation ultimately contributes to the reliability and credibility of the research outcomes.

### Statistical analysis

Statistical analysis was carried out by an independent evaluator with extensive experience in data analysis (S.H.), who was intentionally blinded to the various experimental procedures and the specific group assignments. This approach was designed to minimize bias and ensure objectivity in the interpretation of the results. By keeping the evaluator unaware of the conditions and groupings, the integrity of the analysis was upheld, allowing for a more reliable and valid evaluation of the experimental data. Numerical data were assessed for normality using visual inspection of the data distribution and the Shapiro-Wilk test. The distribution of the bacterial count data was non-parametric and exhibited a marked positive skewness. To account for the skewness, the data underwent a log transformation. Levene’s test revealed a violation of the assumption of homogeneity of variances; thus, the analysis included a robust one-way ANOVA and the Games-Howell post hoc test. The results were reported as mean and standard deviation values. The significance level was set at 5% (R Statistics, version 4.1.3 for Windows, Bell Laboratories, Murray Hill, NJ, USA).

## Results

**Fig. 1:**
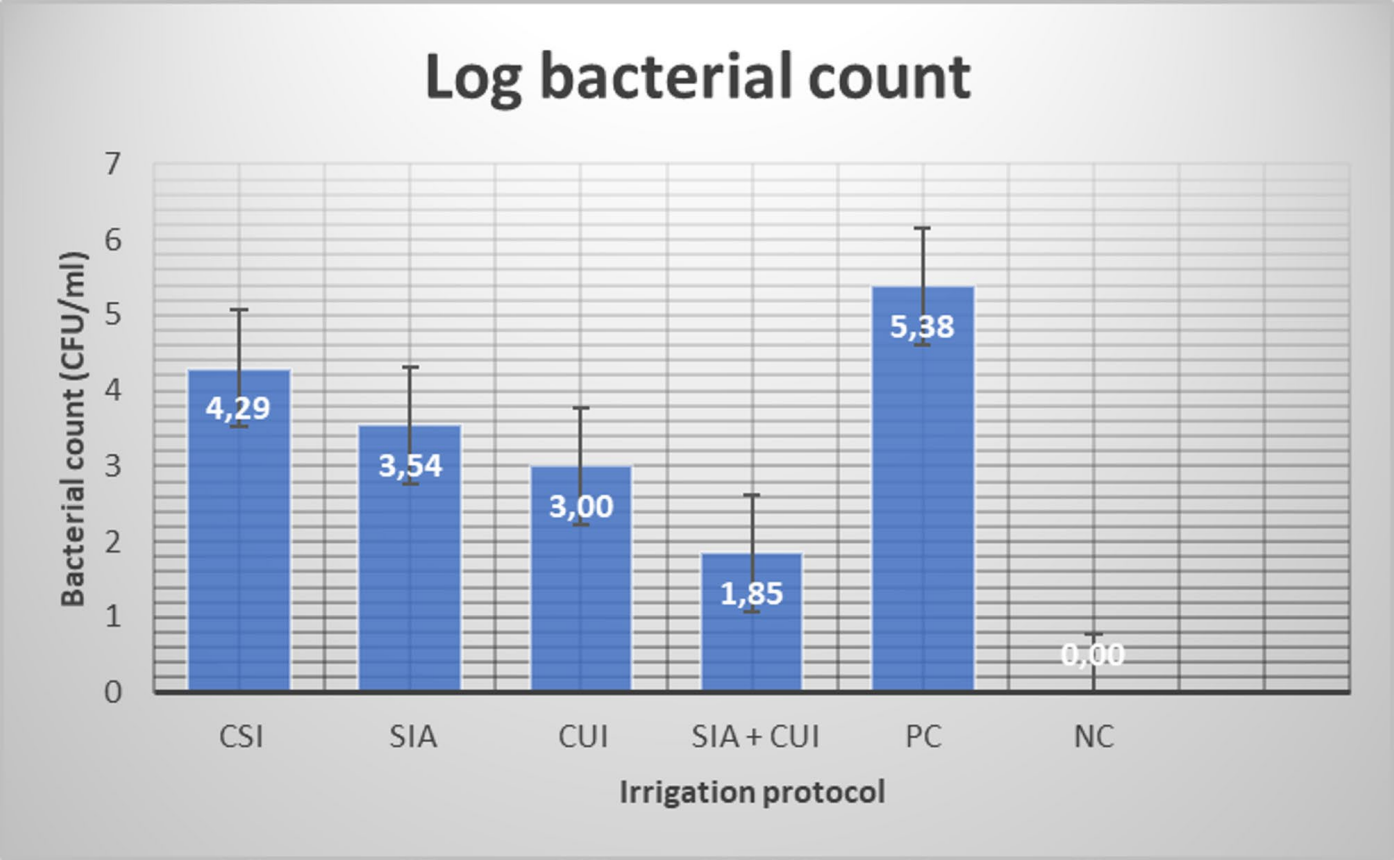
Bacterial count results according to the experimental groups ([CSI] conventional syringe irrigation; [SIA] stepwise intraoperative activation; [CUI] conventional ultrasonic irrigation; [SIA + CUI] stepwise intraoperative activation plus conventional ultrasonic irrigation, [PC] positive control; [NC] negative control groups)

The present study involved a detailed comparative analysis of four distinct experimental groups (*n* = 10 each), each representing a unique irrigation strategy designed to assess their disinfecting effectiveness under controlled conditions. In addition to these experimental groups, two control groups (*n* = 10 each) were established to serve as benchmarks for evaluation. This design allows for a thorough examination of the implications and outcomes associated with each irrigation strategy, facilitating a comprehensive understanding of their impacts on the disinfection of oval root canals. The mean and standard deviation of the log bacterial count (CFU/mL) for the different irrigation protocols are summarized in Table 1 and illustrated in Fig. 1. Although none of the tested irrigation protocols was able to eradicate the intracanal bacterial infection completely, all of them significantly reduced the bacterial load (*p* <.001). When the different irrigation protocols were compared, the combination of SIA and CUI exhibited the highest statistically significant bacterial reduction (1.85 ± 0.99), followed by the CUI (3.00 ± 0.13), SIA (3.54 ± 0.26), and CSI (4.29 ± 0.16) groups, respectively with statistically significant differences between them (*p* <.05) (Table 2). The aseptic procedures were confirmed by the absence of bacterial growth in the negative control group (uncontaminated samples) (0.00 ± 0.00). In contrast, the positive control group ensured bacterial viability and growth throughout the experiment (5.38 ± 0.05).

**Table 1:** The mean and standard deviation of the log bacterial count (CFU/ml) for the different irrigation protocols

Irrigation protocol groups	Bacterial count (CFU/ml) (means ± standard deviation)	*p* value
Conventional syringe irrigation (CSI)	4.29 ± 0.16^a^	< 0.001
Stepwise intraoperative activation (SIA)	3.54 ± 0.26^b^	
Conventional ultrasonic irrigation (CUI)	3.00 ± 0.13^c^	
Stepwise intraoperative activation + Conventional ultrasonic irrigation (SIA + CUI)	1.85 ± 0.99^d^	
Positive control (PC)	5.38 ± 0.05^e^	
Negative control (NC)	0.00 ± 0.00^f^	

Superscript letters indicate significant differences

Significance was set at < 0.05

**Table 2:** Multiple comparisons between different groups

Irrigation protocol groups	Comparison groups	The mean difference between groups	Standard error	*p* value
Conventional syringe irrigation (CSI)	SIA	0.74334	0.09778	< 0.001
	CUI	1.27802	0.06500	< 0.001
	SIA + CUI	2.44157	0.31782	< 0.001
	PC	-1.08858	0.05365	< 0.001
	NC	4.28751	0.05149	< 0.001
Stepwise intraoperative activation (SIA)	CSI	− 0.74334	0.09778	< 0.001
	CUI	0.53468	0.09211	0.001
	SIA + CUI	1.69823	0.32445	0.003
	PC	-1.83192	0.08448	< 0.001
	NC	3.54417	0.08313	< 0.001
Conventional ultrasonic irrigation (CUI)	CSI	-1.27802	0.06500	< 0.001
	SIA	− 0.53468	0.09211	0.001
	SIA + CUI	1.16355	0.31612	0.040
	PC	-2.36660	0.04244	< 0.001
	NC	3.00949	0.03967	< 0.001
Stepwise intraoperative activation + Conventional ultrasonic irrigation (SIA + CUI)	CSI	-2.44157	0.31782	< 0.001
	SIA	-1.69823	0.32445	0.003
	CUI	-1.16355	0.31612	0.040
	PC	-3.53015	0.31398	< 0.001
	NC	1.84594	0.31362	0.002
Positive control (PC)	CSI	1.08858	0.05365	< 0.001
	SIA	1.83192	0.08448	< 0.001
	CUI	2.36660	0.04244	< 0.001
	SIA + CUI	3.53015	0.31398	< 0.001
	NC	5.37609	0.01507	< 0.001
Negative control (NC)	CSI	-4.28751	0.05149	< 0.001
	SIA	-3.54417	0.08313	< 0.001
	CUI	-3.00949	0.03967	< 0.001
	SIA + CUI	-1.84594	0.31362	0.002
	PC	-5.37609	0.01507	< 0.001

## Discussion

Our results demonstrate that while none of the irrigation protocols were capable of rendering the oval root canals free from infection, all the irrigation approaches considerably reduced the intra-radicular bacterial burden. The comparative analysis exhibited a clear hierarchy in disinfection efficacy: The combination of SIA and CUI achieved the greatest bacterial reduction, followed by the CUI, SIA, and CSI, respectively, with significant differences between them. Thus, the null hypothesis was rejected.

Despite the remarkable surge witnessed in the development of mechanical instruments, large percentages of the canal walls remain untouched during mechanical instrumentation [40]. It has been reported that these untouched areas harbor a large amount of pulp tissue remnant, debris, and bacterial biofilms [5, 16], which, in turn, may compromise treatment outcomes, particularly in infected cases [41]. This issue is exacerbated in oval root canals, where the buccolingual dimension is twice the mesiodistal dimension [42–44]. In these cases, mechanical file systems, regardless of type, often tend to create a centered circular preparation, leaving non-instrumented buccal and lingual extensions or recesses [42–44]. Therefore, effective supplemental disinfecting protocols must be implemented to enhance the role of mechanical instruments in disinfecting oval canals.

Irrigation is considered an integral part of root canal preparation to effectively clean and disinfect areas unreached by mechanical instruments [27]. Syringe irrigation remains the most popular method for delivering irrigants inside root canals [27]. However, the influence of the conventional syringe method typically does not extend beyond 1 mm beyond the tip of the irrigating needle [45]. Consequently, some areas in the root canal remain inadequately cleaned and disinfected, which may jeopardize treatment success [45]. As root canals are considered closed-ended cavities, air bubbles can become trapped in their apical end, creating a phenomenon known as apical vapor lock [46]. This obstruction limits the deep penetration of irrigants, compromising debridement and disinfection of the apical root canal [46]. Another limitation of conventional syringe irrigation is its inability to eliminate apical vapor lock [47]. To improve the flow and diffusion of irrigants and maximize their effectiveness, especially in complex anatomies such as oval root canals, activation of the root canal irrigant has been suggested [48, 49].

Despite the diversity of techniques employed for irrigation activation, passive ultrasonic irrigation remains the standard and most popular method for irrigant activation [27]. Traditionally, passive ultrasonic irrigation has been suggested as a supplemental approach to enhancing the effectiveness of basic chemo-mechanical preparation through the activation of irrigants at the end of root canal procedures [50, 51]. This process creates acoustic streaming and cavitations that force the irrigant to apply more shear stresses on the canal wall, disrupting attached debris, pulp remnants, and bacterial biofilms [50, 51]. Additionally, it can eliminate the apical vapor lock that impedes effective disinfection in the apical part [26]. Furthermore, it has been speculated that passive ultrasonic activation raises the irrigant’s temperature, subsequently increasing its chemical reactivity, which promotes greater antibacterial action and tissue dissolution [52–54]. Also, the resultant heating of the irrigant may decrease its viscosity, hence increasing its flow to more distant areas [53]. Despite all of these advantages, it remains challenging to completely eradicate bacteria, especially in complex root canal configurations like oval canals, which raises the risk of treatment failure [11, 12]. This necessitates ongoing research for more adjunctive regimens to maximize disinfection in such intricate configurations.

Ultrasonic stepwise intraoperative irrigant activation, which involves the activation of NaOCl between shaping files, was first introduced in endodontic literature by Plotino et al. [28] to enhance the irrigation protocol. So far, research has demonstrated that such a regimen exhibited promising results regarding root canal debridement [28], while there is no scientific data available concerning its antimicrobial efficacy, particularly in oval root canals. Thus, the current study aimed to compare the disinfecting capability of stepwise intraoperative activation of NaOCl alone and in conjunction with conventional ultrasonic activation to the conventional syringe irrigation protocol in oval canals.

The use of non-biological models, like resin root canals, as substrates for biofilm formation has a number of benefits, such as standardizing structure and composition and addressing individual differences in dentine composition, age, and traits. Furthermore, compared to opaque biological materials, the transparency of these models makes it possible to monitor the activity of the tools and irrigation solutions as well as the eradication of biofilms more clearly. However, compared to dentine, biofilm adhesion and development in these artificial models differ significantly. According to reports, dentine offers a good substrate for the attachment and maturation of different microbial species in addition to mimicking a real-world clinical scenario [55]. Moreover, the structure of the dentine might act as a buffer against the effects of substances like NaOCl, reducing its action [56]. Given all these factors, the present ex-vivo study was conducted on extracted teeth rather than other non-biological materials, mimicking the natural environment of biofilm adherence and growth.

The prevalence of root canals with simple round cross-sections is considered an exception rather than a rule [7]. Most of the root canals clinicians encounter in routine clinical practice possess varying degrees of ovality [57]. As the degree of ovality increases, root canal debridement and disinfection become more challenging [57]. Single-rooted maxillary second premolars with single root canals are one of the most common tooth types to have oval and long oval root canals, with a buccolingual dimension twice or four times the mesiodistal width, respectively [8, 58]. That’s why maxillary second premolars with single oval root canals were selected in the present study.

Although decoronation seems feasible for assessing root canal microbiota [31], access cavities were performed in the current study to replicate the clinical scenario, serving as a reservoir for irrigants in addition to their impact on subsequent root canal procedures [39, 59].

Conventional access cavities were established in the current investigation to secure adequate access for introducing the instruments and irrigants inside the root canals without interferences or incidences of procedural errors that may occur with contracted access cavities [59].

Automated rotary systems offer several advantages over manual techniques, although manual instrumentation is still commonly used by practitioners. These advantages include faster procedures [60], more centered preparations with less canal transportation [61], and less apical debris extrusion [62]. Scientific research has not shown significant differences between engine-driven reciprocating file systems and those that use continuous rotation motions regarding root canal disinfection [63]. However, the latter has demonstrated the ability to remove root canal contents in a more coronal direction, as opposed to reciprocating systems that tend to pack them more laterally on the canal walls [18, 43, 64]. Therefore, root canals in the present study were instrumented using an engine-driven file system with continuous rotation motion.

Despite the wide disparity in endodontic literature regarding the efficiency of disinfecting root canals, particularly those with oval cross-sections, using multi-file or single-file engine-driven systems [14, 31, 39, 44, 64–67], multi-file systems offer the advantage of multiple insertions of more than one file inside the root canal, with subsequent multiple angles of insertion. This may result in more interactions with larger surface areas of the canal walls, while single-file systems are associated with much fewer insertions [67]. Additionally, multi-file systems allow the use of irrigants in larger volumes and for longer durations than single-file systems, enhancing the mechanical flushing effect, antimicrobial effectiveness, and tissue-dissolving capability of the root canal irrigants [27, 67]. Given these considerations and to corroborate preceding observations, which illustrated that engine-driven instruments in conjunction with manual instrumentation might optimize oval root canal disinfection [17, 18], the present investigation used a multi-file rotary system boosted with manual circumferential filing of the buccal and lingual extensions of the root canal using a stainless-steel H file.

Even though research has not yet reached a meaningful conclusion regarding the ideal apical preparation size [68], larger apical sizes are more recommended, particularly in infected root canals, for several reasons [69–73]. Firstly, it has been advocated that mechanical instruments play a pivotal role in bacterial reduction [13, 14]. Secondly, enlargement of the apical preparation allows deeper insertion of the irrigating needle inside the root canal to overcome the viscosity of the irrigants, allowing their deeper penetration and hence increasing the effectiveness of root canal irrigation, along with avoiding binding of the needle to permit the backflow of the irrigant with subsequent enhanced mechanical flushing action, in addition to the exchange of the irrigant [27, 74, 75]. Thirdly, as previously mentioned, larger preparation is associated with copious amounts of irrigants [31, 67]. Fourthly, larger apical preparations reduce the likelihood of irrigant extrusion [76]. On the other hand, the taper of the root canal preparation seems to be less important for the penetration of irrigation into the apical part [77]. Taking all these considerations into account and being in line with Prado et al. [78] who reported that apical enlargement in premolar teeth up to size 45 may reduce their resistance to fracture, root canals in the present study were prepared up to size 40/0.04. This allowed deeper passive insertion of the used 30-gauge side-vented closed-end irrigating needle, which corresponds to size 30, and the ultrasonic tip used for irrigant activation, which corresponded to a size 20/0.01, up to 1 mm and 3 mm from the working length, respectively, to ensure effective irrigation protocols [75, 76, 79].

Consistent with a substantial body of literature supporting the use of side-vented closed-end irrigating needles to prevent accidental irrigant extrusion, particularly when NaOCl is used in higher concentrations [27, 75, 76], the current study utilized a side-vented, closed-end irrigating needle despite the higher efficiency of the open-ended needles in cleaning and disinfecting the apical part [80]. NaOCl was used in full concentration, although there is no consensus on a particular concentration, to align with the reported tendency towards using higher concentrations for more effective disinfection and soft tissue dissolution [47, 81].

Despite its indisputable antimicrobial capabilities, unique organic tissue dissolving ability, and its role as a lubricant for mechanical instruments, NaOCl must be supplemented with a chelating agent like EDTA to address its main flaw in dissolving inorganic substances and consequently enhance the removal of the smear layer, which may harbor a considerable amount of microorganisms [47, 82, 83]. Additionally, EDTA has been reported to augment the anti-biofilm action of NaOCl, despite its weak antimicrobial effect, through the detachment of the biofilm structure from the canal walls [84, 85]. Thus, while NaOCl is usually used during instrumentation alone, it should be adjunct with EDTA as a final rinse following the completion of root canal instrumentation [82]. The present study followed preceding reports that the alternate use of 10 mL of 5.25% NaOCl and 10 mL of 17% EDTA [86], each for 2 min [82], has been suggested for more effective disinfection and smear layer removal. Considering the reported detrimental effects on dentin structure that might result from the use of EDTA prior to NaOCl [87] or the dire sequelae of combining both irrigants [47, 82], the standard irrigation protocol employing NaOCl irrigation between instruments and a final rinse of alternate use of 10 mL of 5.25% NaOCl for 2 min followed by 10 mL of 17% EDTA for 2 min, with intermediate irrigation using a saline solution with the same volumes and time, was accomplished in the current study. Additionally, saline was also used for the final flush to prevent the unremitting EDTA-induced softening of the canal walls [47].

When the irrigant is ultrasonically activated, there are two approaches: continuous or intermittent passive ultrasonic activation. When oscillation is started and stopped repeatedly, it improves cleaning efficacy and may aid in biofilm removal more effectively than when the activation is done continuously for the same duration [36, 88]. Additionally, frequent replenishment of the irrigant compensates for irrigant loss from splashing out of the pulp chamber [51] and its consumption in chemical reactions [89]. Therefore, the use of intermittent ultrasonic activation was preferred in this study. To obtain the maximum benefits from the ultrasonic activation of the irrigant, the inserted instrument must lie passively inside the root canals to avoid breakage, cutting into the canal wall, and producing stronger acoustic streaming and cavitations. These are the reasons behind the replacement of the former concept of “active ultrasonic activation” with “passive ultrasonic irrigation“ [49]. It has been observed that the smaller the activated ultrasonic file or tip, the more powerful acoustic streaming and cavitations are generated within the irrigant, exerting greater shear stresses on the canal walls and eliminating the apical vapor lock [27, 47]. Therefore, in this study, an ultrasonic tip of size 20/0.01 was used and inserted passively up to 3 mm short of the working length [27, 47].

Contrary to the Plotino et al. study [28], the stepwise intraoperative ultrasonic activation of NaOCl in the current investigation used a fresh solution instead of the one injected by the plastic syringe between the instruments for two main purposes: firstly, to increase the volume and exposure time to NaOCl, thereby enhancing its antimicrobial and tissue dissolution capacities [47, 82]; and secondly, to mitigate the rapid chlorine consumption [27, 56].

Among the diverse range of endodontic infections, *E. faecalis* stands out for its resistance to chemo-mechanical preparation and various disinfectants, its ability to survive in tough environments, and its association with most endodontic failures [90, 91]. Furthermore, it is easier to culture and manipulate [92]. Therefore, *E. faecalis* species were selected for the current study and sampled using the culture method with paper points, which provides a simple and reliable way to determine bacterial load and virulence factors [39, 93, 94]. Similar outcomes have been reported when comparing the polymerase chain reaction approach to the culture method [39]. This has encouraged numerous previous studies to utilize the culture method [39, 65, 95–98]. In an effort to bolster the validity of the results in the current study, a small-sized H file was employed in a pumping motion (an in-and-out motion) along all the canal walls to allow more disruption of the attached biofilms prior to culturing with paper points [14, 31, 96, 98].

Corroborating the overwhelming evidence [65, 99–101], our findings confirmed that chemo-mechanical preparation, regardless of the irrigation protocol employed, reduced the bacterial count significantly without completely eliminating the infection.

The current study affirmed the vital role of irrigation in eliminating bacteria from the root canal system. While the mechanical flushing effect of irrigation has been found to reduce the bacterial load [102], it has been suggested that the irrigant should have antibacterial properties to ensure optimal canal disinfection [99]. This helps explain the results of the present study, in which even conventional syringe irrigation aided in significant bacterial reduction.

When comparing different irrigation protocols, the present study demonstrated that stepwise intraoperative activation of NaOCl combined with conventional ultrasonic irrigation achieved the highest statistically significant intracanal bacterial reduction. This was followed by conventional ultrasonic irrigation, stepwise intraoperative activation of NaOCl, and conventional syringe irrigation, respectively, with statistically significant differences between all the tested irrigation methods. The significant superiority of the combination of both activation approaches over the other methods could be explained by the higher delivered volumes, longer irrigation times, more cycles of irrigant activation, and the resulting increase in acoustic streaming, cavitation, and internal heating of the irrigants, which enhance the potential to eliminate the apical vapor lock.

It has been noted that as the root canal is enlarged as much as possible, passive ultrasonic activation becomes more effective with stronger acoustic streaming and cavitation [27]. This might help explain why, when both activation techniques were compared separately, conventional ultrasonic irrigation reduced the bacterial burden more effectively than stepwise intraoperative irrigant activation.

In line with previous research [26, 96, 103], the current investigation revealed that the passive ultrasonic activation of NaOCl significantly increased bacterial reduction compared to the conventional syringe protocol. Conversely, other studies [99, 104, 105] were unable to identify any discernible difference between the two methods. These discrepancies might be attributed to different samples and study designs.

Our findings showed that stepwise intraoperative activation of NaOCl significantly reduced the bacterial load compared to the conventional syringe technique. This might be explained by the larger volumes and longer irrigation duration, which enhance the effectiveness of NaOCl. Additionally, the resultant acoustic streaming, cavitation, and internal heating of the irrigants, along with the subsequent elimination of the apical vapor lock due to the intraoperative activation of NaOCl, contribute to its increased effectiveness.

The findings of the current study suggest that stepwise intraoperative activation of NaOCl can enhance the disinfection of oval root canals considerably, with additional significant improvement achievable when combined with conventional passive ultrasonic activation. Furthermore, maximal disinfection of oval root canals appears to be the cumulative and concerted effect of using a multi-file system with an adequate (larger) apical preparation size, manual circumferential filing of the buccal and lingual extensions, larger volumes, and longer contact time of antimicrobial root canal irrigant delivered through a deeply seated small diameter irrigating needle, and the combined effect of both stepwise intraoperative NaOCl activation and conventional passive ultrasonic irrigation activation.

The findings of this ex-vivo investigation can hold significant implications for clinicians who are tasked with managing anatomically intricate root canal systems, especially those featuring oval-shaped canals. These unique canal configurations frequently result in considerable sections of the canal walls being inaccessible to instruments, which hampers the efficacy of mechanical debridement as a standalone treatment method. Our research has highlighted the advantages of employing a dual approach that combines stepwise intraoperative irrigation activation with final ultrasonic activation. This innovative strategy was shown to substantially enhance disinfection within these challenging canal systems. The improvement is likely due to a more effective exchange of irrigants and the disruption of the vapor lock that can otherwise hinder fluid flow in the complex apical anatomical spaces. By integrating these supplementary techniques into routine clinical practice, clinicians can enhance the removal of biofilm from canal walls, which is critical for effective disinfection. This comprehensive methodology not only may reduce the risk of persistent infections but also promote higher success rates in endodontic treatments involving complex canal anatomies. Overall, our results advocate for a paradigm shift in the approach to treating difficult root canal systems, with a focus on more thorough and reliable disinfection strategies.

### Limitations, strengths, and future perspectives

The major limitation of the current study lies in its ex vivo design. Indeed, Ex-vivo studies come with specific limitations that need careful consideration when interpreting the results. One significant constraint is that these tests are performed under highly controlled laboratory conditions, which may not accurately replicate the complexity of in vivo environments. This includes factors such as variations in individual patient biology, including genetic differences, immune response variations, and the influence of comorbid conditions that can affect how tissues or cells behave in real-world scenarios. Consequently, while ex-vivo studies can reveal important mechanisms and effects, one must be cautious in generalizing these findings to patient populations without accounting for these individual nuances.

The intricate and polymicrobial nature of root canal infections presents significant challenges in the treatment of these conditions. A notable constraint of the current investigation is the reliance on monospecies biofilms, which may not accurately represent the clinical scenarios typically encountered in practice. In real-world contexts, root canal infections are characterized by a complex interplay of diverse microorganisms. These organisms not only exist simultaneously but also engage in various interactions that can complicate the infection landscape. Despite this limitation, the use of single-species biofilms offers important advantages. Specifically, researchers benefit from a heightened level of standardization and better control over experimental variables, which significantly enhances the reproducibility of findings in laboratory settings when compared to multi-species biofilms. Introducing multiple bacterial species into biofilm models introduces a series of complexities. Chief among these challenges is the uneven distribution of bacteria across different samples, which can stem from varying environmental preferences and growth rates among distinct bacterial taxa. Such diversity can affect their ability to coexist and thrive within a mixed-species biofilm, resulting in varied interactions and behaviors that are difficult to study and standardize. Moreover, the inherent complexity of multi-species biofilms can hinder the reproducibility of experimental outcomes, complicating interpretations and making valid comparisons challenging. In light of these considerations, a monospecies biofilm model was selected for the present experiment. This approach enables researchers to maintain stricter control over experimental conditions and better understand the behaviors and interactions of individual bacterial strains. However, to enhance the generalizability and relevance of the findings, future studies should aim to incorporate diverse multi-species biofilm models. Such investigations are critical for fully capturing the intricate dynamics present in real-world root canal infections and for advancing both the scientific understanding and clinical management of these cases.

Additionally, the field currently lacks a universally recognized gold standard for assessing root canal disinfection. Each assessment method has its own strengths and limitations. Culture methods may fail to detect microorganisms residing in inaccessible areas of the root canals, overlook viable but non-culturable (VBNC) bacteria, and neglect the assessment of residual biofilm structures. To improve evaluation accuracy, we suggest incorporating techniques such as quantitative polymerase chain reaction (q-PCR), fluorescence-based viability assays, and sophisticated imaging modalities like confocal laser scanning microscopy. Moreover, combining these methodologies will facilitate a more thorough and nuanced evaluation of disinfection efficacy. Therefore, further research that integrates these diverse assessment techniques is essential to advance our understanding of root canal disinfection and improve clinical outcomes for patients suffering from root canal infections.

Despite all these limitations, the present investigation possesses a few strengths. Firstly, the study introduced a novel approach to enhance disinfection in intricate morphologies like oval root canals. Secondly, the present experiment aimed to provide research with a trustful clinical translation to routine clinical practice. It has been demonstrated that laboratory studies must hold high relevance to clinical endodontic practice to yield robust benefits for clinicians and patients alike. In an important effort to enhance the validity and reliability of the results obtained from this laboratory-based research to impeccable levels, further well-designed prospective randomized clinical trials are needed to assess the impact of such an emerging concept on clinical and radiographic outcomes. Thirdly, to avoid conducting underpowered research, not only did the current investigation determine the total sample size which was 30 (5 per group) based on a sample size calculation with a power of 95%, but was also carried out using a larger sample size of 60 (10 per group) that represented the double of the pre-determined sample size to better detect true differences between groups and to provide more trustworthy results. Considering the promising outcomes of stepwise intraoperative NaOCl activation in terms of disinfection, as reported in the present study, and debridement [28], the complete removal of root canal filling materials during root canal retreatment represents another challenging issue in daily clinical practice. Therefore, future studies are required to evaluate the effectiveness of such a technique in removing obturating materials.

## Conclusion

Within the constraints of the present study, it is reasonable to conclude that stepwise intraoperative activation of NaOCl can improve the disinfection capacity of the chemo-mechanical preparation in oval root canals. Maximal disinfection might be achieved when boosted with conventional passive ultrasonic activation. Nevertheless, this conclusion was drawn under the specific conditions of the current research. Thus, to enhance the generalizability and reliability of the present findings, it is essential to conduct further studies with an adequate sample size across diverse laboratory and clinical settings, and variations in root canal morphology, as well as multi-modal biofilm assessment approaches to provide a more holistic grasp of the influence of this novel approach on root canal disinfection.

This manuscript has been meticulously crafted in accordance with the Preferred Reporting Items for Laboratory studies in Endodontology (PRILE 2021) guidelines. These comprehensive standards serve as a robust framework designed to enhance transparency and rigor in reporting laboratory studies specifically related to endodontics. By adhering to these guidelines, the integrity and reproducibility of the research are significantly bolstered, allowing for greater trust in the findings.

**Fig. 2:**
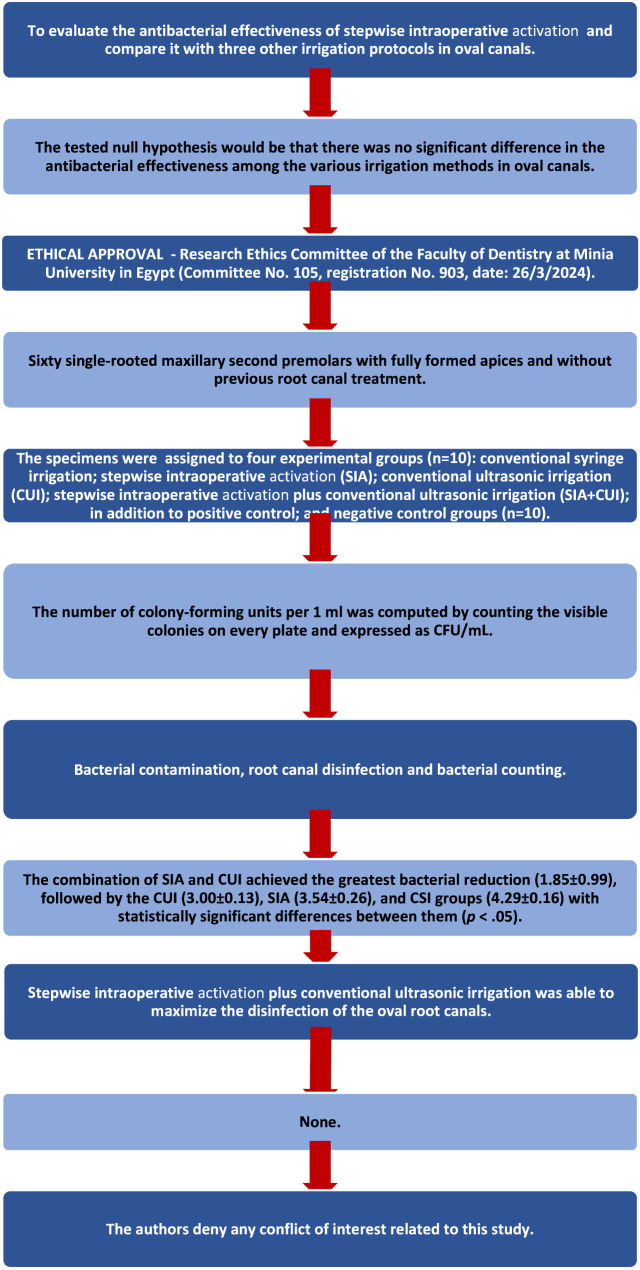
PRILE 2021 flowchart. *From Nagendrababu V, Murray PE, Ordinola-Zapata R, Peters OA, Rôças IN, Siqueira JF Jr, Priya E, Jayaraman J, Pulikkotil SJ, Camilleri J, Boutsioukis C, Rossi-Fedele G, Dummer PMH (2021) PRILE 2021 guidelines for reporting laboratory studies in Endodontology: a consensus-based development. International Endodontic Journal May 3. doi: 10.1111/iej.13542. https://onlinelibrary.wiley.com/doi/abs/10.1111/iej.13542. For further details visit: http://pride-endodonticguidelines.org/prile

The results obtained from the current laboratory study are succinctly presented in the PRILE 2021 flowchart, which is provided as Fig. 2. This flowchart delineates the critical elements of the study, such as the overarching purpose of the research, the formulated null hypothesis, detailed methodology, significant results, and concluding insights. By visually representing these components, the flowchart greatly enhances comprehension, allowing readers to grasp the intricate research process and the implications of the outcomes more effectively.

## Electronic supplementary material

Below is the link to the electronic supplementary material.

Supplementary Material 1

## Data Availability

All data or materials generated or analyzed during this study are included in this article.
